# From Person to Patient: Power, Control and the Construction of Vulnerability in the Operating Room

**DOI:** 10.1111/nin.70179

**Published:** 2026-10-20

**Authors:** Mirko Prosen

**Affiliations:** ^1^ Faculty of Health Sciences University of Primorska Izola Slovenia

**Keywords:** clinical practice, interprofessional relations, organisational dynamics, patient experience, perioperative care

## Abstract

The operating room is a highly organised clinical environment in which patients experience a shift from autonomous individuals to dependent recipients of care. This transformation is often regarded as a clinical necessity, but it is also actively shaped by everyday practices, organisational structures and professional interactions. This study explored how healthcare professionals interpret mechanisms of power, control and patient vulnerability in the operating room. A qualitative design was used, employing semi‐structured interviews with 32 healthcare professionals (13 perioperative nurses, 6 nurse anaesthetists and 13 surgeons) from a general hospital. Data were analysed using reflexive thematic analysis. Three themes were identified: (1) From person to patient: loss of control and transformation in the operating room; (2) The body as object: routinised practice and negotiated authority; and (3) Ethics under pressure: dignity and vulnerability in everyday practice. The findings show that patient vulnerability is not only a consequence of clinical condition but also actively produced through routine practices, hierarchical relations and organisational priorities. Anaesthesia emerged as a critical turning point, where attention shifts from the person to the procedure. Although these practices are often normalised as necessary for efficiency and safety, they may contribute to reduced patient visibility and agency.

## 1 Introduction

The operating room is widely regarded as a highly controlled and technologically advanced clinical environment, where precision, efficiency and patient safety are prioritised. However, such a perspective risks overlooking its fundamentally social and organisational character. The operating room represents a complex interactional space in which healthcare professionals engage not only in technical tasks but also in a range of social practices, including cooperation, informal communication, teaching and conflict (L. K. Jones et al. 2018). Precisely because the operating room prioritises safety, technical precision and procedural coordination, it provides an important setting for examining how patient vulnerability is organised and managed in clinical practice. Dependence on healthcare professionals, anaesthesia, physical exposure and workflow demands shape how patients are positioned within care, while professional roles, institutional norms and hierarchical structures influence both team functioning and the management of patient vulnerability (Amini Rarani 2025; Etherington et al. 2021; L. K. Jones et al. 2018).

Communication within the operating room is central to the coordination of care, yet opportunities to contribute to clinical communication are not equally distributed among team members. Qualitative studies of interprofessional operating room teams have shown that professional hierarchy shapes who speaks, when concerns are raised and how openly team members question decisions or communicate perceived risks (Etherington et al. 2021; Gardezi et al. 2009). Nurses, for example, may face constraints in expressing concerns or questioning decisions, particularly in interactions with surgeons, reflecting entrenched professional hierarchies (Gardezi et al. 2009). Although these dynamics primarily shape team interactions, they have direct implications for the patient. In this context, effective communication involves the timely exchange of clinically relevant information, the clear articulation of concerns and the ability of team members to advocate for patient safety and needs across professional boundaries.

Communication also encompasses more than verbal exchange. Silence, manifested as hesitation, omission or absence of response, plays a significant role in shaping team dynamics and may limit the articulation of concerns related to patient care. Rather than indicating passivity alone, silence may reflect underlying power relations, including fear of negative evaluation, adherence to professional boundaries or strategic adaptation to hierarchical expectations (Brommelsiek et al. 2021; Gardezi et al. 2009). Furthermore, communication preferences within surgical teams may reinforce these dynamics, as patterns of interaction are shaped by role expectations and authority structures (Reeder et al. 2025). Consequently, both speech and silence not only organise team interaction but also shape how patient needs, risks and vulnerabilities are recognised and addressed within the operating room. In this sense, communication practices do not merely transmit information but actively shape how patient vulnerability is interpreted, articulated and responded to within everyday clinical practice (C. Jones and Durbridge 2016).

The organisation of communication reflects broader hierarchical and relational dynamics that structure clinical practice. Patterns of cooperation and conflict are closely linked to professional roles and authority, with tensions often arising between individuals in different hierarchical positions (L. K. Jones et al. 2018). These dynamics are embedded in routine practices and not limited to overt disagreements. Even seemingly minor decisions, such as control over environmental factors like music, can reveal underlying distributions of authority, with surgeons more likely to assume decision‐making control than other team members (Lamberton et al. 2023). Such practices illustrate how control is exercised in subtle and routine ways, shaping not only team interactions but also influencing which aspects of patient care are prioritised, how concerns are addressed and to what extent the patient's needs are actively considered within the operating room.

From a theoretical perspective, these dynamics can be understood through a Foucauldian framework, in which power is conceptualised as relational, productive and embedded in everyday practices rather than possessed solely by individuals. In clinical settings, power operates through disciplinary mechanisms that regulate behaviour, define professional roles and establish norms of appropriate conduct (Ristić et al. 2021; Suijker 2023). In the operating room, these mechanisms are evident in communication practices, expectations of role adherence and the maintenance of hierarchical order. Practices such as silence, deference to authority and adherence to established routines function as forms of disciplinary power that structure how healthcare professionals act and interact (Brommelsiek et al. 2021; Gardezi et al. 2009). Foucault's concept of the medical gaze further illuminates how the patient is positioned within this environment. The patient is understood primarily through clinically relevant bodily and procedural dimensions rather than as a whole person (Foucault 2003). Such objectification is produced through institutional practices and professional discourses that prioritise efficiency, technical performance and control (Suijker 2023).

In this study, power is understood as a broader organisational and relational phenomenon operating through norms, hierarchies, routines and professional expectations, whereas control refers to the concrete enactment of these dynamics in everyday clinical practice, particularly through workflow coordination, task management and decision‐making (Nimmon and Stenfors‐Hayes 2016). Within the operating room, control serves as a necessary organisational and clinical mechanism for maintaining patient safety, procedural coordination and effective interdisciplinary collaboration. At the same time, it is exercised through hierarchical relationships that shape whose perspectives influence decision‐making and how patient vulnerability, dignity and autonomy are managed in perioperative practice (Gardezi et al. 2009; L. K. Jones et al. 2018; Ristić et al. 2021).

These organisational and interpersonal dynamics have direct implications for the patient, who occupies a uniquely vulnerable position within the operating room. Patients undergoing surgery experience a transition from autonomy to dependence, characterised by physical exposure, uncertainty and reliance on healthcare professionals. Research has shown that perioperative patients often experience loss of control, feelings of insecurity and depersonalisation in this highly technical environment (Cousley 2015; Sutton et al. 2022). Importantly, patient vulnerability should not be understood solely as an individual condition but as something shaped and intensified by the organisational context, communication practices and power relations that structure the operating room (Istanboulian et al. 2026). Within the context of operating room, patient‐centred care may not primarily involve active participation in decision‐making during the procedure itself, but rather the preservation of dignity, personhood, respectful communication and recognition of vulnerability within highly technical forms of care (Leonardsen et al. 2024; Zhou et al. 2025).

Ethical considerations are inherently embedded within these dynamics. The operating room requires continuous balancing between efficiency, technical performance and respect for patient dignity, autonomy and privacy. Ethical challenges frequently arise when hierarchical structures, communication constraints or time pressures influence decision‐making and interactions (Jeon et al. 2024). Ethical practice therefore cannot be reduced to adherence to formal principles alone but must be understood within the broader system of power, control and vulnerability that shapes clinical care and the patient's experience.

Despite increasing attention to communication, teamwork and patient‐centred care, existing research remains fragmented. Studies have examined communication dynamics (Gardezi et al. 2009; Reeder et al. 2025), social interactions and hierarchy (Brommelsiek et al. 2021; C. Jones and Durbridge 2016), environmental and decision‐making practices (Lamberton et al. 2023), ethical issues (Jeon et al. 2024) and patient vulnerability (Cousley 2015; Sutton et al. 2022) largely in isolation. There is limited integration of these perspectives into a coherent understanding of how power and control are enacted in everyday practice and how they shape patient vulnerability within the operating room. The aim of this study is to explore how healthcare professionals in the operating room experience and interpret mechanisms of power, control and patient vulnerability.

## 2 Methods

### 2.1 Study Design

This study adopted an interpretive qualitative design informed by a Foucauldian theoretical perspective. Foucauldian concepts of power, control, subjectivity and the medical gaze provided a theoretical lens for examining how professional practices, organisational structures and social relations shape patient vulnerability within the operating room (Foucault 2003). Accordingly, the study sought not only to describe participants' experiences but also to interpret how meanings and practices surrounding power, control and vulnerability are produced and negotiated within everyday clinical work. Data were analysed using reflexive thematic analysis, as developed by Braun and Clarke (2006, 2019, 2021).

### 2.2 Participants and Setting

The Slovenian healthcare system is universal and predominantly publicly funded, based on mandatory social health insurance, ensuring near‐universal population coverage. Healthcare delivery is organised across three levels: primary care, provided by general practitioners and community health centres acting as gatekeepers; secondary care, comprising specialist outpatient services and hospital care, and tertiary care, delivered by highly specialised institutions, including university medical centres. Hospital provision is largely publicly owned and includes approximately 30 institutions, consisting of two university medical centres, around 10 general hospitals and various specialised facilities such as psychiatric and rehabilitation hospitals (Marušič and Prevolnik Rupel 2021; Ministry of Health 2025). This study was conducted at a secondary‐level teaching general hospital in Slovenia, which provides a broad range of surgical services across multiple specialties. The operating department comprised multidisciplinary perioperative teams, including surgeons, anaesthesiologists, nurse anaesthetists, perioperative nurses and nursing assistants, working in a highly coordinated and time‐sensitive clinical environment.

Participants were recruited using purposive sampling to ensure the inclusion of healthcare professionals with direct experience of perioperative work and familiarity with the organisational, interpersonal and hierarchical dynamics of the operating room. The sampling strategy focused on three professional groups, perioperative nurses, nurse anaesthetists and surgeons, representing distinct roles and responsibilities within perioperative care. The final sample comprised 32 healthcare professionals: 13 perioperative nurses, 6 nurse anaesthetists and 13 surgeons.

### 2.3 Data Collection

Eligible participants were invited to take part on a voluntary basis and were provided with information about the purpose of the study, confidentiality, voluntary participation and their right to withdraw. All participants who agreed to take part provided written informed consent before data collection. Before the interviews, participants completed a brief sociodemographic form. Data were collected through individual, face‐to‐face, semi‐structured interviews conducted in hospital settings, in locations where privacy and confidentiality could be ensured and interruptions minimised. The interview guide was developed by the researcher and informed by a theoretical perspective drawing on Foucauldian concepts of power, control and subjectivity, which guided both the structure of the interviews and the exploration of participants' experiences. Two versions of the interview guide were developed for nurses (Supplement S1) and surgeons (Supplement S2). While the guides were largely comparable in thematic structure, minor adaptations were made to account for differences in professional roles, responsibilities and positions within the clinical hierarchy. This enabled the study to capture how power relations and clinical practices are experienced and enacted differently across professional groups, while maintaining analytical coherence across the dataset.

Interviews were conducted in December 2025, lasted approximately 45 minutes on average and were audio‐recorded with participants' permission. Sampling was guided by the concept of information power, whereby the adequacy of the sample was determined by the relevance and richness of the data in relation to the study aim, rather than by a predefined sample size. In line with this approach, recruitment continued until the data provided sufficient depth and variation to address the research question. To support this process, field notes and reflective writing were completed after each interview, enabling ongoing assessment of data richness and analytical relevance.

### 2.4 Data Analysis

All audio recordings were transcribed verbatim and checked against the original recordings to ensure accuracy. The transcripts were then imported into NVivo version 14 (Lumivero 2022) for qualitative data analysis. Quantitative data, including sociodemographic characteristics, were analysed using IBM SPSS Statistics version 29 (IBM Corp 2023).

Data were analysed using reflexive thematic analysis, following the six‐phase approach proposed by Braun and Clarke (2006, 2021). The analysis was iterative and reflexive, involving close engagement with the data through repeated reading, coding and the development of subthemes and themes through continuous comparison and interpretation. Themes were not considered to ‘emerge’ from the data but were actively constructed through the researcher's engagement with the dataset, consistent with a reflexive approach to thematic analysis (Braun and Clarke 2021). The researcher has a professional background in nursing and academic experience in healthcare research, which provided familiarity with clinical environments and interprofessional practice. The researcher was not employed in the study setting and had no direct professional or supervisory relationship with the participants. This position offered contextual understanding but also carried the potential for prior assumptions about professional roles, hierarchy and patient care to shape data generation and interpretation. As the sole researcher, particular attention was given to reflexivity throughout the research process, including critical consideration of how these assumptions might influence data interpretation. Reflexive notes were recorded after each interview to capture initial impressions and analytic insights.

As part of the reflexive analytic process, emerging interpretations and developing themes were discussed with two academic colleagues experienced in qualitative research and familiar with the topic. These discussions were not intended to establish coding consensus or validate a single ‘correct’ interpretation of the data, but to provide opportunities for critical dialogue, challenge taken‐for‐granted assumptions and consider alternative interpretations during theme development. Consistent with the study's interpretive orientation, the analysis moved beyond predominantly semantic meanings towards a more latent and interpretive engagement with the data, examining how participants' accounts reflected underlying assumptions, professional norms, organisational arrangements and broader relations of power within the operating room. Foucauldian concepts were used as sensitising theoretical resources rather than as a predetermined coding framework, allowing themes to be developed through reflexive engagement between the data, the researcher and the theoretical perspective. Detailed documentation of the analytic process was maintained to provide transparency regarding the development of the final themes.

### 2.5 Ethical Considerations

Ethical approval for the study was obtained from the institutional ethics committee prior to data collection (Commission of the University of Primorska for Ethics in Human Subjects Research; Approval No. 4264‐16‐3/2022). All participants were informed about the purpose of the study and their rights, including voluntary participation, the right to withdraw at any time and the option to decline answering any questions without consequences. Written informed consent was obtained from all participants. Data were handled in accordance with applicable data protection regulations, including the General Data Protection Regulation (GDPR). To ensure confidentiality and protect participants' identities, all quantitative data were anonymised and reported in aggregated form, which was particularly important given the single‐centre nature of the study. Audio recordings were deleted after transcription.

## 3 Results

### 3.1 Participants Characteristics

The study included 32 healthcare professionals: 13 perioperative nurses, 6 nurse anaesthetists and 13 surgeons. The sample comprised 21 women (65.6%) and 11 men (34.4%), representing a broad range of ages and professional experience. Perioperative nurses had a mean age of 42.1 years (SD = 11.3; range 24–59) and an average of 17.1 years (SD = 12.1) of professional experience in healthcare, including 10.5 years (SD = 8.7) specifically in perioperative nursing. Nurse anaesthetists had a mean age of 36.8 years (SD = 10.1; range 26–52) and an average of 14.0 years (SD = 11.6) of healthcare experience, including 7.3 years (SD = 6.9) in anaesthesia care. Surgeons had a mean age of 43.7 years (SD = 7.8; range 30–54) and an average of 16.5 years (SD = 7.5) of healthcare experience, including 15.3 years (SD = 8.1) in surgical practice. Across professional groups, working arrangements included predominantly daytime schedules, as well as shift‐based work and on‐call duties, including 24‐hour shifts. Participants had experience in a range of surgical specialties, including general, urological, gynaecological, orthopaedic, thoracic, vascular, trauma and otorhinolaryngological surgery, reflecting diverse perioperative clinical experience.

### 3.2 Thematic Structure of Operating Room Experience, Practice and Ethics

The thematic analysis identified three overarching themes: (1) From person to patient: loss of control and transformation in the operating room; (2) The body as object: routinised practice and negotiated authority, and (3) Ethics under pressure: dignity and vulnerability in everyday practice (Table 1).

**Table 1:** Overview of themes and subthemes.

Theme	Subthemes
From person to patient: Loss of control and transformation in the operating room	Entering the operating room: Fear, exposure and loss of control
	Anaesthesia as a turning point: From person to procedure
The body as object: Routinised practice and negotiated authority	Working on the body: Routine, detachment and task orientation
	Power in practice: Hierarchy and negotiated authority within the team
Ethics under pressure: Dignity and vulnerability in everyday practice	Dignity under pressure: Ideals versus organisational reality
	Normalising vulnerability as part of routine care

#### 3.2.1 From Person to Patient: Loss of Control and Transformation in the Operating Room

The theme ‘From person to patient’ describes how participants across professional groups described the patient's entry into the operating room as a moment characterised by exposure, uncertainty and a gradual loss of control. The operating room was consistently depicted as a space that disrupts the patient's sense of autonomy, where familiar social roles are suspended and replaced by dependence on an unfamiliar team.

Perioperative nurses often described this moment through a relational perspective, emphasising the patient's isolation and the abrupt transition into an environment filled with unknown professionals. The patient was portrayed as ‘alone among strangers,’ required to entrust their body and safety to individuals they had not previously met. This loss of control was both physical and social, as patients entered a space governed by rules and routines that were not transparent to them.

I see them as very alone, surrounded by people they don't know… they have to surrender to us.

(Perioperative nurse)

This sense of uncertainty and loss of control was also evident in nurse anaesthetists' accounts, although they placed greater emphasis on the emotional and sensory aspects of this transition. The presence of masked staff, technical equipment and rapid procedural preparation contributed to a sense of disorientation and dependence. As nurse anaesthetists noted, patients experience ‘a loss of control and complete reliance on the team,’ further intensified by the anonymity of the clinical environment.

They come in anxious, looking around, trying to understand what is happening… but everything is unfamiliar, everyone is covered, and they realise very quickly that they have no control over the situation anymore.

(Nurse anaesthetist)

Surgeons also recognised this fear and uncertainty, but tended to frame it within the expected trajectory of surgical care. Fear and uncertainty were recognised yet positioned as transient and secondary to the procedural flow. Compared to nurses, surgeons were less likely to focus on the relational or emotional aspects of the encounter, instead situating the patient's experience within a broader clinical sequence.

Of course they are afraid, that is normal… but we see it's part of the process. The focus is on getting them safely through the procedure, not on that initial fear.

(Surgeon)

Across all professional groups, anaesthesia was described as a critical turning point that formalised the patient's transition from an active participant in interaction towards a more passive object of clinical care. Participants described this as the moment when the patient ‘lets go completely,’ relinquishing both control and the capacity to participate in what follows. For nurse anaesthetists, this moment was particularly significant, representing both a technical intervention and a relational threshold in which responsibility for the patient is fully assumed by the team.

The greatest fear comes just before losing consciousness, when they completely hand themselves over to us.

(Nurse anaesthetist)

This transition was also evident in perioperative nurses' accounts, although they described the shift primarily in relation to changes within the team itself. Once the patient is anaesthetised, attention shifts decisively towards technical tasks, positioning and coordination, and the atmosphere in the room changes. One perioperative nurse observed that while the team remains quieter when the patient is awake, ‘once the patient is asleep, everything becomes faster, louder, more task‐focused.’ In this sense, the patient's loss of consciousness also marks a reorganisation of priorities within the team.

When the patient is still awake, we are more careful, more attentive to them as a person… but the moment they fall asleep, the focus shifts completely. Then it's about instruments, positioning, timing. Everything at that moment becomes more mechanical, more about the procedure than the person.

(Perioperative nurse)

Surgeons described the same transition in more explicitly functional terms, linking it to the need for precision, efficiency and clinical focus. Emotional distancing was not presented as problematic but as necessary, enabling the surgeon to engage fully with the procedure. As one surgeon explained, the patient becomes ‘a case’ or ‘a task,’ allowing the work to proceed without distraction.

At that point, you have to switch your mindset. You can't think of them as a person lying there – you have to think in terms of the procedure, the steps, the risks. Otherwise, you lose focus. It becomes a case you need to solve.

(Surgeon)

Across all professional groups, the patient's transformation was described not as a single event but as a process shaped by the interaction of environmental conditions, professional practices and organisational routines. While all professional groups recognised the patient's vulnerability, perioperative nurses and nurse anaesthetists more often emphasised its experiential and relational dimensions, whereas surgeons tended to situate it within the functional logic of clinical work. Rather than being a deliberate shift, this transformation appeared as something embedded in the everyday organisation of care, where participants described the patient as gradually becoming redefined from a person with agency to a body requiring intervention.

It's not that we stop seeing them as a person on purpose… but everything is organised in a way that, at some point, they simply become the body we are working on.

(Perioperative nurse)

#### 3.2.2 The Body as Object: Routinised Practice and Negotiated Authority

The second theme ‘The body as object’ reflects how clinical work in the operating room is organised around the patient's body as the central object of intervention, shaped by routinised practices and embedded within hierarchical, yet dynamically negotiated, team structures. Across all professional groups, participants described a shift towards task‐oriented work in which the body becomes the primary focus of attention, structured through protocols, timing and coordination.

Perioperative nurses described their work as highly structured and repetitive, organised around sequences of tasks that must be performed with precision and efficiency. Within this framework, the patient's body was approached primarily through its procedural requirements, positioning, preparation, exposure and instrument handling, rather than as a holistic entity. This routinisation was not described as optional but as necessary to maintain safety and workflow continuity.

You don't really think about the whole person at that point… you focus on the field, the instruments, what needs to be done next. Everything follows a sequence, and you just move from one step to another.

(Perioperative nurse)

At the same time, their accounts demonstrated an awareness of this reduction, often implicitly acknowledging the tension between technical focus and person‐centred care. Their accounts suggested that objectification is not a deliberate act but an outcome of the organisational logic of the operating room.

You are aware that this is a person, but at the same time you have to focus on the task… and sometimes that part – the person – just fades into the background because the work demands it.

(Perioperative nurse)

This tension was therefore understood not simply as an individual response, but as embedded in the organisation of clinical work.

It's not that you want to treat them as an object, but everything is organised in a way that pushes you in that direction.

(Perioperative nurse)

A similar orientation towards the body was evident in nurse anaesthetists' accounts, although their focus remained closely tied to physiological monitoring and control. The patient was primarily encountered through vital signs, parameters and responses to anaesthetic agents, reinforcing a form of clinical attention centred on measurable bodily functions. In this sense, the body was an object not only of intervention but also of continuous surveillance.

You follow the numbers, the reactions, the parameters… that's how you know what's happening. The patient becomes what you see on the monitor and how the body responds.

(Nurse anaesthetist)

Surgeons articulated this orientation most explicitly, framing the body as the direct site of technical work. Their accounts emphasised precision, visual focus and the need to isolate the operative field, often reducing the body to specific anatomical structures relevant to the procedure. This narrowing of attention was described as essential for effective performance.

You concentrate on the operative field… that's your world at that moment. Everything else becomes irrelevant – you focus on the structure, the tissue, what needs to be corrected.

(Surgeon)

Alongside this object‐oriented work, participants across groups described the operating room as a strongly hierarchical environment, in which authority is formally structured yet practically negotiated. Surgeons were consistently positioned at the top of this hierarchy, associated with decision‐making power and overall responsibility for the procedure. However, this authority was not absolute and was often described as dependent on the smooth functioning of the team. A perioperative nurse described this dynamic as formally clear but practically interdependent:

There is a clear hierarchy, the surgeon leads, but in reality, things only work if everyone contributes and adjusts to each other.

(Perioperative nurse)

This negotiated character of authority was also reflected in the accounts of nurse anaesthetists, who emphasised more subtle forms of influence within the hierarchical structure:

Sometimes you don't challenge directly, but you find ways to influence what is happening… it's more subtle than just following orders.

(Nurse anaesthetist)

Surgeons, however, tended to frame this structure more in terms of coordination and responsibility than negotiation:

In the end, someone has to take responsibility, and that's the surgeon: but of course, it only works if the whole team functions well.

(Surgeon)

Across professional groups, the narratives indicate that the operating room functions as a highly organised setting in which the patient's body is approached as the focus of coordinated clinical work, structured through routines, technologies and professional roles. Within this setting, authority appears both formally defined and practically negotiated in everyday interactions. While perioperative nurses and nurse anaesthetists more frequently described the adjustments and coordination required in this process, surgeons tended to frame it in terms of efficiency, control and procedural focus.

Everything is organised around the body and the task, and everyone has their place in that, but how things actually unfold depends on how we work together in the moment.

(Perioperative nurse)

In the end, someone has to take responsibility, and that's the surgeon. The decisions come from there.

(Surgeon)

#### 3.2.3 Ethics Under Pressure: Dignity and Vulnerability in Everyday Practice

The last identified theme ‘Ethics under pressure’ illustrates how participants across professional groups described the tension between maintaining patient dignity and managing the practical demands of everyday work in the operating room. While respect for the patient was consistently identified as a core professional value, participants also highlighted the constraints imposed by time pressure, workload and organisational routines, which often limited the extent to which dignity could be actively upheld in practice.

Perioperative nurses most frequently framed this tension in relational and ethical terms, emphasising the importance of preserving the patient's dignity even in highly technical and time‐sensitive situations. They described efforts to protect the patient's privacy, maintain respectful communication and minimise unnecessary exposure, while acknowledging that these practices are not always fully achievable.

You try to cover them, to speak to them respectfully, to keep some sense of dignity… but sometimes the pace is too fast, and not everything is done the way you would ideally want.

(Perioperative nurse)

These accounts suggest that dignity is not a stable condition but something that must be actively negotiated within the constraints of the clinical environment. This negotiation was also evident in nurse anaesthetists' accounts, particularly in relation to the induction of anaesthesia and the immediate preoperative phase. They described patients as highly dependent and often unable to fully comprehend or control what is happening, which places an additional ethical responsibility on the team. However, they also acknowledged that this vulnerability becomes less visible once the patient is anaesthetised.

Before they fall asleep, they are very vulnerable – they look at you, they depend on you completely… but once they are under anaesthesia, that awareness somehow disappears, and the focus shifts.

(Nurse anaesthetist)

Surgeons, in contrast, framed dignity within the broader goal of successful surgical outcomes, often positioning technical competence and procedural efficiency as primary expressions of professional responsibility. While they did not deny the importance of respectful treatment, their accounts suggested that dignity is safeguarded indirectly through effective clinical work rather than through explicit relational practices.

For me, dignity means doing the operation well and safely… that is the most important thing for the patient in that situation.

(Surgeon)

Beyond these different professional understandings of dignity, all professional groups described how exposure of the patient's body is a routine and unavoidable aspect of surgical care, yet one that carries ethical implications. While efforts are made to limit unnecessary exposure, participants acknowledged that the demands of access, visibility and efficiency often take precedence.

The body is exposed because it must be. That's part of the procedure… you try to limit it, but you cannot avoid it completely.

(Perioperative nurse)

This necessity was framed in more explicitly procedural terms by surgeons:

You need proper access and visibility to do the operation safely… sometimes that means more exposure, but it's not unnecessary. It's what allows you to do the job correctly.

(Surgeon)

Repeated exposure to such situations was also described as contributing over time to a process of normalisation. What might initially be perceived as ethically sensitive or uncomfortable becomes integrated into routine practice, reducing emotional responsiveness and allowing work to proceed more efficiently.

At the beginning, it affects you more… but with time, you get used to it. It becomes part of the job, something normal.

(Nurse anaesthetist)

Participants described this normalisation not necessarily as negative, but rather as a functional adaptation that enables professionals to perform their roles within a demanding environment. At the same time, some participants expressed concern that this process may distance them from the patient's experience and reduce sensitivity to issues of dignity and vulnerability.

You get used to it because otherwise you couldn't do the job… but at the same time, you know that this also makes you less sensitive to what the patient might be experiencing.

(Nurse anaesthetist)

Across participants' accounts, ethical considerations in the operating room were embedded within a context shaped by competing priorities, where maintaining dignity was often contingent on situational demands rather than consistently enacted. Rather than being fully upheld or neglected, dignity appeared as something fluid and context‐dependent, negotiated in real time within the constraints of clinical work. In this sense, vulnerability was not only recognised but also gradually integrated into routine practice, becoming an expected and, to some extent, normalised feature of the operating room environment.

You know they are vulnerable, but you also know that you have a job to do… and somehow, you learn to balance that, even if it's not always perfect.

(Perioperative nurse)

## 4 Discussion

This study offers an integrated understanding of how patient vulnerability in the operating room is not simply an individual or situational experience but is actively shaped by organisational structures, professional practices and interactional dynamics. By examining healthcare professionals' perspectives across different roles, the findings show that power, control and vulnerability are deeply interconnected and embedded in the everyday functioning of the operating room. Rather than existing as abstract concepts, they are enacted through routine practices, communication patterns and the organisation of work, collectively shaping how the patient is positioned within this highly technical environment.

A central contribution of this study is the conceptualisation of the transformation from person to patient as a dynamic and processual phenomenon. While previous research has described perioperative vulnerability mainly in terms of patients' experiences of anxiety, exposure and loss of control (Cousley 2015; Sutton et al. 2022), the present findings extend this understanding by showing how such vulnerability is actively produced through clinical routines and environmental conditions. The transition begins upon entry into the operating room, where patients encounter an unfamiliar setting characterised by technological complexity, masked professionals and limited opportunities for interaction. This process intensifies with anaesthesia, which emerges as a critical turning point that formalises the patient's shift from active participant to passive recipient of care. Rather than occurring as a single moment, this transformation appeared to unfold progressively through a series of interconnected relational, organisational and procedural transitions. Participants described how unfamiliar surroundings, masked professionals, technical preparation, changes in communication patterns, induction of anaesthesia and the subsequent shift towards task‐oriented coordination collectively contributed to the patient's gradual repositioning from an active social subject to a clinically managed body. While the present findings illuminate how healthcare professionals interpret and enact patient vulnerability, an important tension arises when these perspectives are considered alongside patient accounts reported in the literature. Patients describe perioperative vulnerability not only as a clinical or situational condition but as a deeply personal and affective experience, characterised by uncertainty, fear and a profound loss of control (Cousley 2015; Meigh et al. 2024; Sutton et al. 2022). This suggests that vulnerability, as constructed within professional practice, may not fully capture how it is lived and experienced by patients themselves. The processes identified in this study, including the gradual transformation of the patient into a passive recipient of care, may therefore contribute not only to organisational efficiency but also to experiences of invisibility, insecurity and reduced personhood. At the same time, patient vulnerability in the operating room cannot be reduced solely to experiences of powerlessness or loss of autonomy. Several participants described situations in which patients seemed willing to entrust control to healthcare professionals, particularly when professional competence, reassurance and emotional presence contributed to feelings of safety. In this sense, dependence within perioperative care may also represent a relational form of trust rather than merely a passive loss of agency (Sellman 2005). Vulnerability therefore emerges as a more complex and context‐dependent condition, shaped not only by exposure to clinical control but also by patients' expectations of protection, expertise and compassionate care (Cousley et al. 2014).

These processes are closely linked to the operation of power within the operating room. Consistent with previous research on hierarchical dynamics and communication constraints (Etherington et al. 2021; Gardezi et al. 2009), the findings indicate that authority is formally structured yet practically negotiated. However, rather than being concentrated solely in specific professional roles, power operates in a relational and diffuse manner, shaping interactions through norms, expectations and routine behaviours. From a Foucauldian perspective, power is embedded in everyday practices that regulate what can be said, who can speak and how actions are performed within the clinical environment (Ristić et al. 2021; Suijker 2023). In this study, such mechanisms are evident not only in overt decision‐making but also in more subtle forms of interaction, including silence, indirect influence and strategic adaptation to hierarchical expectations. Healthcare professionals are thus not merely constrained by power structures but actively participate in their reproduction through everyday practices, including how they communicate, how they remain silent and how they position themselves within the team (Gardezi et al. 2009).

Closely related to power is the concept of control, which can be understood as its practical enactment in specific clinical situations. In the operating room, control is exercised through the coordination of tasks, management of workflow and prioritisation of technical objectives (Göras et al. 2020). The findings highlight how control becomes particularly visible following the induction of anaesthesia, when attention shifts decisively from the patient as a person to the procedural demands of the operation. This shift is accompanied by changes in tempo, communication style and team interaction, reflecting a reorganisation of priorities within the team. Previous studies have similarly emphasised the role of professional roles and authority in shaping decision‐making and interaction patterns (Dahmani et al. 2024; Göras et al. 2020; L. K. Jones et al. 2018), as well as the presence of subtle forms of control in everyday practices (Lamberton et al. 2023). The present study builds on previous work by demonstrating how such practices not only structure team functioning but also directly influence how the patient is perceived, engaged with and ultimately reduced to a clinical object within the surgical process.

Another important finding concerns the normalisation of these practices within clinical work. Participants across professional groups did not necessarily view the transformation of the patient into a more objectified form as problematic, but rather as a functional adaptation required to ensure efficiency, precision and safety. This normalisation reflects a broader organisational logic in which technical performance and procedural flow are prioritised (Aouicha et al. 2022). At the same time, some participants expressed awareness of the potential consequences of this process, particularly regarding distancing from the patient and reduced sensitivity to issues of dignity and vulnerability. This tension highlights a significant ethical dimension of operating room practice. As noted in previous research, ethical challenges in this setting often arise not from explicit violations but from routine practices shaped by hierarchical structures, time pressure and organisational demands (Agledahl and Pedersen 2024; Jeon et al. 2024). From a Foucauldian perspective (Foucault 2003), such dynamics can be understood as processes of normalisation, through which certain practices become taken for granted and rarely questioned, even when they may have implications for patient experience.

### 4.1 Implications

The findings of this study have several important implications for clinical practice. Efforts to promote patient‐centred care in the operating room cannot rely solely on formal protocols or guidelines but must also address the implicit practices and relational dynamics that shape everyday work. In particular, there is a need to move beyond general awareness towards structured opportunities for reflexive practice, enabling healthcare professionals to critically examine how routine behaviours, including silence, task prioritisation and forms of objectification, may influence patient experience (Kearns et al. 2021).

The findings also highlight the importance of maintaining the patient's personhood throughout the perioperative process. This may involve deliberate practices such as verbal engagement prior to anaesthesia, continued acknowledgement of the patient during procedures and increased team awareness of the patient as a person rather than solely as a surgical case. Importantly, these issues cannot be addressed solely at the level of individual behaviour. Organisational structures, including workflow demands, time pressure and hierarchical norms, play a critical role in shaping clinical practice. Interventions should therefore also target organisational culture, including leadership approaches, training programmes and policies that explicitly prioritise patient dignity alongside technical efficiency.

Particular attention should be given to critical transition points, especially the induction of anaesthesia, which participants described as representing not only a clinical intervention but also a shift in the patient's position from an active participant in interaction towards a more passive recipient of care. These moments offer opportunities for targeted interventions aimed at preserving patient dignity and visibility within the clinical process. In this context, the findings point to important ethical implications. Respect for patient dignity in the operating room cannot be assumed but must be actively enacted through everyday practices (Agledahl and Pedersen 2024). Ethical care therefore involves continuous attention to how power, control and routine interactions shape the patient's experience of vulnerability.

### 4.2 Strengths and Limitations

This study has several strengths. It offers an in‐depth qualitative exploration of an under‐researched topic, integrating perspectives from multiple professional groups within the operating room. By focusing on the interplay between power, control and vulnerability, it offers a more comprehensive understanding of how patient experience is shaped within highly technical clinical environments.

However, certain limitations should be acknowledged. The study was conducted within a single institutional context, and the transferability of the findings may be influenced by local organisational culture, team structures and institutional norms, which may differ across settings. In addition, the data are based exclusively on healthcare professionals' accounts and do not include patients' perspectives. Although professionals can provide important insights into practices and interactions that may shape patient vulnerability, they cannot fully capture how vulnerability is experienced and understood by patients themselves. This limitation is particularly important given evidence that patients may experience vulnerability in ways not fully reflected in professional interpretations (Sutton et al. 2022). Future studies should therefore bring patients' and healthcare professionals' perspectives into dialogue, as these may provide complementary insights into how vulnerability is experienced, interpreted and shaped through perioperative practices and interactions.

The study focused on three professional groups and did not include anaesthesiologists or nursing assistants. Their absence limits the range of professional perspectives represented and may have left some dimensions of professional authority, interprofessional relations and patient vulnerability unexplored. This should be considered when interpreting the findings, which reflect the perspectives of the professional groups included rather than those of the wider operating room team.

The reliance on self‐reported data also represents a limitation, as it does not capture how power and control are enacted in real‐time interactions and may differ from participants' descriptions. Furthermore, participants may have been influenced by social desirability and professional norms when discussing sensitive issues such as hierarchy, power and objectification, potentially leading to partial or moderated accounts of practice. At the same time, the interpretation of findings was informed by a Foucauldian perspective, which may have shaped the analytical focus on power and control, potentially foregrounding certain aspects of the data while placing less emphasis on alternative interpretations. Such an approach could be complemented by theoretical perspectives that foreground emotion, embodiment and intersubjectivity in perioperative care. As with all qualitative research, the findings reflect interpretative analysis and are influenced by both participant accounts and the analytical lens applied.

## 5 Conclusion

The study shows that the operating room does not simply provide technical care but actively constructs the conditions in which patients are transformed from individuals with agency into clinical objects of intervention. Power and control are not confined to formal hierarchies but are embedded in routine practices, interactions and organisational norms that structure daily work. Patient vulnerability, therefore, arises not only from clinical condition but also from these practices, raising important questions about how care is organised and how patient dignity is maintained in highly technical environments.

## Author Contributions

**Mirko Prosen:** conceptualization, methodology, investigation, data curation, formal analysis, writing – original draft, writing – review and editing, visualization, supervision, project administration.

## Funding

The author has nothing to report.

## Ethics Statement

Ethical approval was obtained from the Commission of the University of Primorska for Ethics in Human Subjects Research (Approval No. 4264‐16‐3/2022).

## Conflicts of Interest

The author declares no conflicts of interest.

## Artificial Intelligence Disclosure

Generative artificial intelligence (AI) was not used for data collection or to independently conduct the qualitative analysis or generate the study findings. The language was reviewed using the InstaText application (non‐AI based), and the manuscript was subsequently professionally proofread by a native English speaker an official capacity.

## Supporting information


Supporting File 1

Supporting File 2

## Data Availability

The datasets used in this study are not available due to ethical restrictions on sharing raw data. However, they can be obtained from the corresponding author upon reasonable request.
