# Supplementary material for: From Person to Patient: Power, Control and the Construction of Vulnerability in the Operating Room
Source: Nurs Inq. 2026 Oct 20;33(4):e70179. doi: 10.1111/nin.70179 (PMC13682684; doi:10.1111/nin.70179)
Supplement: Supplementary file 1 [file NIN-33-e70179-s001.docx]

**INTERVIEW GUIDE**

**INTRODUCTION**

Thank you for agreeing to participate in this study. The interview will take approximately 45 to 60 minutes. Everything discussed today will be treated as strictly confidential. Your identity will not be disclosed, and all personal data will be anonymised. If you mention names of colleagues, patients, or specific departments, this information will be removed from the analysis.

You are free to withdraw from the study at any time without any consequences or explanation.

The purpose of this research is to explore how healthcare professionals in the operating room perceive situations in which patients are particularly vulnerable. We are interested in your observations regarding how team relationships, role distribution, and everyday working conditions influence patient care. The study aims to understand how patient dignity, exposure, and safety are perceived during surgical procedures.

**OPENING – Warm-up**

1. How would you describe a patient at the moment they enter the operating room?
2. What do you consider to be the most stressful or vulnerable aspect for the patient at this moment?

**MAIN PART**

3. How do you experience the final moments before anaesthesia, when the patient is still conscious?
4. In your opinion, what changes most significantly when the patient loses consciousness and the ability to communicate?
5. How would you describe relationships among team members when making decisions during surgery?
6. How do you perceive your responsibility and role as the leading member of the team?
7. Do situations arise where decisions must be made rapidly without broader consultation? How does this affect team dynamics?
8. What aspects of communication with the surgical and anaesthesia team support smooth workflow?
9. What do you see as the greatest barriers to communication during procedures?
10. How do you respond when a team member raises a concern or suggests an alternative approach?
11. In which situations do you most strongly feel that the patient is exposed or vulnerable as a person, not merely as a “case”?
12. How do you attempt in practice to preserve patient dignity (e.g., covering, positioning, tone of communication in the operating room)?
13. Have you experienced situations where technical demands or procedural speed took precedence over attention to the patient as a person? Can you provide an example?
14. Have you ever faced a dilemma where a decision could affect the patient’s dignity or safety?
15. How do you see the surgeon’s role in shaping a culture of respect and safety in the operating room?

**CLOSING**

16. In your opinion, what could be improved in the organisation of work to reduce patient exposure and increase protection?
17. What can the operating team as a whole do to ensure more coordinated and patient-centred care?
18. Is there anything else you would like to add?

**CONCLUSION**

Thank you for your time, openness, and willingness to share your experiences. Your insights will contribute to improving ethical, safe, and sensitive patient care in the operating room.

If you would like to receive feedback on the study results, you may let me know now or at any later time.

Thank you again for your participation.
