# Supplementary material for: From Person to Patient: Power, Control and the Construction of Vulnerability in the Operating Room
Source: Nurs Inq. 2026 Oct 20;33(4):e70179. doi: 10.1111/nin.70179 (PMC13682684; doi:10.1111/nin.70179)
Supplement: Supplementary file 2 [file NIN-33-e70179-s002.docx]

**INTERVIEW GUIDE**

**INTRODUCTION**

Thank you for agreeing to participate in this study. The interview will take approximately 30 minutes. Everything discussed today will be treated as strictly confidential. Your identity will not be disclosed, and all personal data will be anonymised. If you mention names of colleagues, patients, or specific departments, this information will be removed from the analysis.

You are free to withdraw from the study at any time without any consequences or explanation.

The purpose of this research is to explore how healthcare professionals in the operating room perceive situations in which patients are particularly vulnerable. We are interested in your observations regarding how team relationships, role distribution, and everyday working conditions influence patient care. The study aims to understand how patient dignity, exposure, and safety are perceived during surgical procedures.

**OPENING – Warm-up**

1. How would you describe a patient when they enter the operating room?
2. What do you perceive as the most difficult or exposing aspect for the patient at this moment?

**MAIN PART**

3. How do you experience the moments just before anaesthesia, when the patient is still conscious and able to communicate?
4. What do you observe in the patient once they fall asleep and can no longer speak or express their needs?
5. How would you describe relationships among members of the operating team—who has the greatest influence in decision-making?
6. How does this hierarchy affect your work and your ability to express your opinion?
7. Have you ever been in a situation where your judgement was not taken into account? How did that make you feel?
8. When do you feel that the patient’s dignity is most at risk?
9. How do you ensure that the patient is appropriately covered, protected, and treated with respect?
10. Have there been situations where, due to time pressure or technical demands, the patient as a person was overlooked? Can you provide an example?
11. Have you ever been in a situation where you wanted to protect the patient but were unable to do so due to circumstances?
12. How do you respond when you observe behaviour or comments that you consider inappropriate towards a patient?
13. How do such situations affect you personally, and how do you cope with them?

**CLOSING**

14. How do you perceive your role in protecting the patient in the operating room?
15. In your opinion, what should change to reduce patient exposure and improve protection?
16. What can healthcare professionals themselves do to better safeguard patient dignity and safety?
17. Is there anything else you would like to add?

**CONCLUSION**

Thank you for your time, openness, and willingness to share your experiences. Your insights will contribute to improving ethical, safe, and sensitive patient care in the operating room.

If you would like to receive feedback on the study results, you may let me know now or at any later time.

Thank you again for your participation.
